# Responsiveness of the Shoulder Pain and Disability Index in patients with adhesive capsulitis

**DOI:** 10.1186/1471-2474-9-161

**Published:** 2008-12-03

**Authors:** Einar Kristian Tveitå, Ole Marius Ekeberg, Niels Gunnar Juel, Erik Bautz-Holter

**Affiliations:** 1 Department of Physical Medicine and Rehabilitation, Ullevål University Hospital, University of Oslo, Oslo, Norway

## Abstract

**Background:**

Instruments designed to measure the subjective impact of painful shoulder conditions have become essential in shoulder research. The Shoulder Pain and Disability Index (SPADI) is one of the most extensively used scales of this type. The objective of this study was to investigate reproducibility and responsiveness of the SPADI in patients with adhesive capsulitis.

**Methods:**

SPADI test-retest reproducibility was estimated by the "intraclass correlation coefficient" (ICC) and the "smallest detectable difference" (SDD). Responsiveness was assessed by exploring baseline and follow-up data recorded in a recently reported clinical trial regarding hydrodilatation and corticosteroid injections in 76 patients with adhesive capsulitis. "Standardized response mean" (SRM) and "reliable change proportion" (RCP) for SPADI were compared with corresponding figures for shoulder range-of-motion (ROM). The relationship between SPADI and ROM change scores was investigated through correlation and linear regression analyses.

**Results:**

Results for test-retest reproducibility indicated a smallest detectable difference of 17 points on the 0–100 scale, and an intraclass correlation coefficient of 0.89. The SPADI was generally more responsive than ROM. Weak to moderately strong associations were identified between SPADI and ROM change scores. According to the regression model, the three variables baseline SPADI, baseline active ROM and change in active ROM together explained 60% of the variance in SPADI improvement.

**Conclusion:**

This study supports the use of SPADI as an outcome measure in similar settings.

## Background

The Shoulder Pain and Disability Index (SPADI) is a self-administered questionnaire consisting of items grouped into pain and disability subscales. Rating is on visual analogue scales, and the means of the two subscales are combined to produce a total score ranging from 0 (best) to 100 (worst). The SPADI was designed to measure the impact of shoulder pathology in terms of pain and disability, for both current status and change over time. The original developers stated the rationale for developing this type of joint-specific instrument: it was expected to measure the impact of specific joint problems more precisely than global health assessment instruments, and also to be better in demonstrating the effect of a treatment directed at one joint only [1]. These properties are closely linked to responsiveness, defined as the ability of an instrument to accurately detect change when it has occurred [2].

Responsiveness of the SPADI has been assessed using retrospective self-assessment of global change as a reference criterion, a study [3] cited by several researchers. Patients were given their baseline responses when follow-up SPADI scores were recorded along with the global rating of change. Then the SPADI change score was compared to the measure of global change, where the patient had rated his shoulder problem as "cured", "improved", "the same" or "worse" compared to his baseline examination. Based on these comparisons, the authors stated that "the SPADI∆ (baseline – follow-up) discriminated accurately between subjects who improved versus those who stayed the same or worsened" [3]. This statement regarding SPADI responsiveness can hardly be expected to apply to settings in clinical trials where more traditional designs are used when gathering follow-up scores. Retrospective methods of computing responsiveness yield little information about the ability of an instrument to detect treatment effects, and they should not be used as a basis for the choice of an instrument for applications to clinical trials [4,5].

SPADI responsiveness has been compared with the responsiveness of other health assessment scales, both global [6-10] and shoulder-specific [8-11]. SPADI is reported to be one of the more responsive scales [12]. It is, however, problematic to make conclusions on responsiveness based on comparisons only with such very similar types of instruments.

A considerable number of shoulder self-report questionnaires have been proposed [13]. The rationale for their employment in research settings must be that there are advantages concerning the properties of the new instruments. This obvious criterion often seems to be ignored. As a consequence, a variety of instruments sometimes makes it a complex task to interpret the results of trials. Furthermore, for most of the shoulder questionnaires, evidence for their validity in various diagnostic groups used in clinical trials is often limited, at best. The SPADI is no exception to this, even though it is one of the shoulder rating instruments that have been most extensively studied [12]. It has also been employed in several clinical trials involving patients with adhesive capsulitis [14-18].

The objective of this study is to investigate reproducibility and responsiveness of the SPADI when evaluating patients with adhesive capsulitis (See Additional file 1 for the Norwegian version [19] of the SPADI). Reproducibility is assessed with a test-retest of presumably "stable" patients. Responsiveness is investigated by using baseline and follow-up scores from a recently reported clinical trial regarding hydrodilatation and corticosteroid injections in patients with adhesive capsulitis [20]. Subjects included in the clinical trial were outpatients attending the Department of Physical Medicine and Rehabilitation of Ullevål University Hospital in the period Dec. 2003 – June 2005. A hydrodilatation procedure including corticosteroids was compared with the injection of corticosteroids without hydrodilatation. Patients were given three injections with two-week intervals, and all injections were given under fluoroscopic guidance. Seventy-six patients were included and groups were compared six weeks after treatment in order to identify potential treatment effects of hydrodilatation. The main inclusion criteria were shoulder pain and reduction of passive ROM in the affected shoulder of 30° or more for at least two out of three glenohumeral movements (flexion, abduction and external rotation).

There are several aspects of responsiveness [2], reflecting the different ways instruments are used in various settings. "Internal" [21] responsiveness statistics refer to the ability to produce statistically significant changes in scores, dependent on the study population and intervention. Interpretation of SPADI "internal" responsiveness figures is facilitated in this study by reporting corresponding figures for shoulder ROM, thereby allowing for head-to-head comparisons of SPADI and a more traditional outcome measure [22] for shoulder capsulitis.

Responsiveness can also be measured in terms of the strength of the relationship between changes in the outcome measure of interest and changes in some external standard, e.g. important clinical variables. This aspect of responsiveness is called "external" responsiveness [21]. Previous researchers have investigated the relationship between shoulder ROM and shoulder scales in cross-sectional analyses [11,23-27], while our aim is to compare change scores. In general, we expect associations of moderate strength. We expect association with SPADI to be stronger for measures of active ROM than for passive ROM, and we expect stronger associations for the disability subscale than for the pain subscale.

## Methods

The regional ethics committee granted ethical approval for the trial. The procedures followed protocol and complied with the Helsinki Declaration as revised in 1983 and current national ethical standards for such studies.

### Translation

Translation of items was based on recommended guidelines [28]. Two teams of translators with Norwegian as their mother tongue made the first forward translations. Two back-translations were then made by professional translators with English as their mother tongue. A committee reviewed the source and final versions. Forward and back-translations of the SPADI revealed no major difficulties and consensus was reached on a preliminary Norwegian version. This version was pre-tested in a group of patients before a final version was available for the present study.

### Measurements

The SPADI is divided into two subscales: a "pain" subscale and a "disability" subscale. The subscales comprise series of 5 items for "pain" and 8 items for "disability", referring to various problems with their shoulder encountered over the last week. Reported scoring procedures vary slightly in different validity studies [1,3,29]. In this study, each item is responded to by a visual analogue scale ranging from "no pain"/"no difficulty", to "worst pain imaginable"/"so difficult required help". Item scores for each section are averaged to produce separate subscale scores ranging from 0 to 100. A SPADI total score ranging from 0 (best) to 100 (worst) is then produced by averaging the two subscale scores. If more than two items of a subscale are not responded to, no SPADI score is calculated. Within-patient comparisons over time are based on items that were scored on both occasions.

SPADI reproducibility in presumably stable patients is assessed by administering the SPADI two times to each patient with a one-week interval. This time interval was chosen because it seemed long enough for the responder to forget previous scoring details, yet short enough to avoid any important change occurring in patients with this long-lasting condition [30,31]. No patient started any new treatment in this one-week period.

Along with SPADI, scores for active and passive shoulder ROM in four different directions were gathered at baseline and follow-up (as part of the clinical trial): abduction (ABD) and flexion (FLE) from neutral, and internal (INT) and external (EXT) rotation at 45° of abduction. For the present study, scores for the four directions were combined to produce overall measures of active (C.AROM) and passive (C.PROM) range-of-motion for each patient. ROM measurements were made according to a pre-specified protocol [32].

### Statistical procedures

Calculation of SPADI reproducibility in stable patients is based on the within-patient standard deviation (sw), derived from a one-way analysis of variance (ANOVA). We report the "smallest detectable difference", defined as SDD = 1.96 sw x √ 2 = 2.77 sw (ref. "repeatability" [33], "minimum detectable change" [6,34]). The difference between two measurements for the same patient is expected to be less than the SDD for 95% of pairs of observations [33]. The calculation of a common standard deviation for the measurements is based on the absence of heteroscedasticity [35]. Heteroscedasticity refers to a situation in which measurement errors are dependent on the size of the various readings. We investigated the relationship between test-retest differences and SPADI means for each patient by using Bland-Altman plots [33].

Reproducibility is also reported by use of the intraclass correlation coefficient (ICC). While SDD refers to the absolute difference between observations, ICC is the correlation between observations [36]. ICC is computed using a one-way ANOVA model (single measures). ICC values can theoretically range from 0 to 1, a high ICC in this case indicating that within-patient differences are small as compared to between-patient variability in the study population.

Group level "internal" responsiveness is analyzed using the "standardized response mean" (SRM) statistic ("efficiency" [37]), defined as the absolute value of mean change (follow-up minus baseline scores) divided by the SD of this change [21,38]. Confidence intervals of the SRM are calculated assuming the change score is normally distributed [39], and the SD of the change is treated as constant for each outcome. SRMs of different measures are compared by the modified jack-knife procedure as described by Angst et al. [40].

"Internal" responsiveness on the individual level is reported in this study by use of the "reliable change proportion" statistic (RCP [6,41]). This is defined as the proportion of patients improving from baseline to follow-up by more than the smallest detectable difference. While the SRM statistic is closely related to the ability to detect statistically significant differences based on group means and between-patient variability, the RCP statistic relates in a similar way to the ability to detect treatment effects in individuals. According to the method described by Davidson and Keating [41], we report confidence intervals for the "reliable change proportions", and compare estimates for the different outcome measures by use of the Cochrane Q test. When calculating RCP for SPADI, the SDD estimate from the SPADI reproducibility substudy of "stable" patients is used. For shoulder ROM, we use SDD estimates obtained in a previously reported study [32] regarding reproducibility of ROM in "stable" patients with adhesive capsulitis.

"External responsiveness" [21] is investigated in this study by calculating Pearson correlation coefficients (*r*) between changes in SPADI and ROM for the affected shoulder. For a more in-depth analysis of the relationship between ROM and SPADI, we also perform multiple linear regression. SPADI improvement from baseline is the dependent variable, independent variables being baseline SPADI, baseline C.AROM and C.AROM change.

All statistical analyses are carried out by using the software package SPSS 13.0 for Windows^® ^(SPSS, Chicago, IL, USA).

## Results

Seventy-six patients were included in the clinical trial [20]. Fourteen of these patients were not able to take part in the SPADI reproducibility substudy for practical reasons. Furthermore, two patients did not respond to a sufficient number of items at retest. Hence sixty patients were available for the SPADI reproducibility analysis. Details for test/retest scores and reproducibility estimates for subscales and SPADI total are given in Table 1. Distributions of SPADI subscale and total scores on both occasions approximate normal distributions according to plots (not shown). Kolmogorov-Smirnov and Shapiro-Wilk tests were non-significant except for the disability scale at retest. Results indicate a "smallest detectable difference" (SDD) of 17 points for the SPADI total score. This means that for approximately 95% of the pairs of observations, the difference between scores was 17 points or smaller. The intraclass correlation coefficient (ICC) was 0.89.

**Table 1:** SPADI test-retest reproducibility (n = 60)

**Item/Subscale**	**Obs. 1**	**Obs. 2**	**SDD**	**ICC**
	**Mean (SD)**	**Mean (SD)**		**(95% CI)**
How severe is your pain:				
01 At its worst?	7.2 (2.6)	7.4 (2.6)		
02 When lying on the involved side?	6.7 (2.5)	6.9 (2.4)		
03 Reaching for something on a high shelf?	7.4 (2.3)	7.7 (1.9)		
04 Touching the back of your neck?	3.9 (3.3)	5.1 (3.1)		
05 Pushing with the involved arm?	5.0 (2.9)	5.3 (3.1)		
Pain score:	60.2 (19)	64.7 (21)	21	0.85 (0.76–0.91)
				
How much difficulty do you have:				
06 Washing your hair?	5.5 (2.9)	5.6 (2.7)		
07 Washing your back?	7.8 (2.3)	8.1 (1.9)		
08 Putting on an undershirt or pullover sweater?	6.4 (2.2)	5.6 (2.3)		
09 Putting on a shirt that buttons down the front?	3.6 (2.6)	3.9 (2.5)		
10 Putting on your pants?	3.9 (3.0)	4.1 (3.0)		
11 Placing an object on a high shelf?	7.8 (2.1)	8.1 (1.7)		
12 Carrying a heavy object of 10 pounds?	5.8 (3.5)	6.2 (3.4)		
13 Removing something from your back pocket?	6.7 (3.0)	6.8 (2.9)		
Disability score:	59.1 (19)	60.3 (19)	20	0.86 (0.78–0.92)
				
SPADI score:	59.7 (17)	62.6 (19)	17	0.89 (0.82–0.93)

SD = between-subject standard deviation, SDD = smallest detectable difference, ICC = intraclass correlation coefficient.

**Figure 1:**
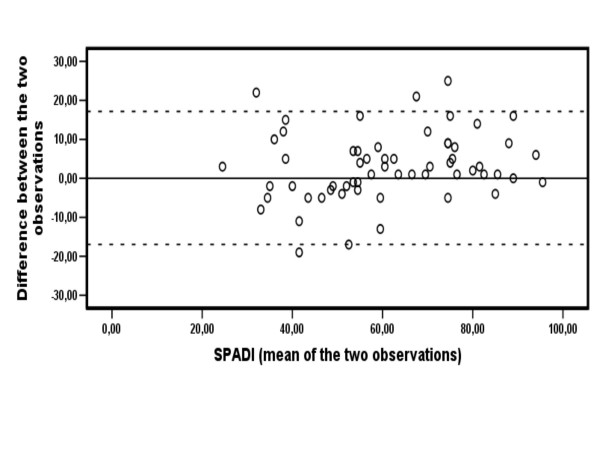
**Test-retest reproducibility: Bland-Altman scatterplot showing mean SPADI value of both observations plotted against the difference between observations for each patient (n = 60)**.

Plotting SPADI means of the two observations against the test-retest difference for each patient does not give any indication that measurement errors vary systematically over the range of possible scores (Figure 1).

### Internal responsiveness

In the responsiveness substudy, all seventy-six patients were included. One of them was not available for follow-up. All other patients responded to a sufficient number of items at follow-up to enable a SPADI score to be calculated. Hence seventy-five patients were available for the responsiveness analysis. Patients in the two treatment arms were pooled as there were no significant differences in treatment effects [20]. Distributions of change scores for ROM and SPADI subscale and total scores approximate normal distributions according to plots (not shown). Kolmogorov-Smirnov and Shapiro-Wilk tests were non-significant except for active abduction (A.ABD) and active flexion (A.FLE).

Results for responsiveness of outcome measures are given in Table 2. According to the modified jack-knife procedure [40], the SPADI total was more responsive than all single-movement ROM measures (p < 0.001 for all these comparisons). The SPADI total was also more responsive than combined ROM (p = 0.01 for C.PROM and p < 0.001 for C.AROM).

**Table 2:** Internal responsiveness of outcome measures (n = 75)

	**Baseline**	**Follow-up**	**Change**	**SRM**	**RCP**
	**Mean (SD)**	**Mean (SD)**	**Mean (SD)**	**(95% CI)**	**(95% CI)**
P. ABD	31.0 (11)	45.0 (13)	14.0 (11)	1.29 (1.06–1.52)	49% (38%–61%)
P. FLE	47.2 (16)	62.9 (13)	15.7 (16)	1.01 (0.78–1.24)	45% (34%–57%)
P. INT	33.0 (13)	47.1 (13)	14.1 (11)	1.29 (1.06–1.52)	47% (35%–58%)
P. EXT	17.4 (14)	27.9 (16)	10.5 (12)	0.85 (0.62–1.08)	33% (22%–44%)
					
A. ABD	55.9 (20)	84.7 (35)	28.8 (32)	0.91 (0.68–1.14)	59% (47%–70%)
A. FLE	88.1 (24)	116.8 (29)	28.7 (29)	0.99 (0.76–1.22)	48% (37%–60%)
A. INT	45.5 (15)	66.9 (17)	21.4 (15)	1.38 (1.15–1.61)	57% (46%–69%)
A. EXT	22.2 (15)	38.1 (19)	15.9 (14)	1.13 (0.90–1.36)	63% (52%–74%)
C. PROM	128.6 (43)	182.9 (44)	54.4 (36)	1.52 (1.29–1.75)	67% (56%–78%)
C. AROM	211.6 (60)	306.5 (88)	94.9 (74)	1.28 (1.05–1.51)	75% (65%–85%)
					
Pain score	62.4 (22)	21.8 (20)	-40.6 (25)	1.62 (1.39–1.85)	77% (68%–87%)
Disability score	60.1 (20)	23.1 (19)	-36.9 (21)	1.76 (1.53–1.99)	77% (68%–87%)
SPADI score	61.3 (20)	22.5 (18)	-38.8 (21)	1.81 (1.58–2.04)	85% (76%–93%)

SD = between-subject standard deviation, CI = confidence interval, SRM = standardized response mean, RCP = reliable change proportion.

When addressing individual-level responsiveness, too, SPADI was more responsive. The Cochrane Q test showed a significantly higher reliable change proportion (RCP) for the SPADI total score than for all single-movement ROM-measures (p < 0.01 for all these comparisons, exact test). The combined ROM measures were generally more responsive than the corresponding single-movement measures, but may be less responsive than the SPADI total.

### External responsiveness

Correlations (*r*) between changes in various ROM scores and SPADI subscale and total scores were in the expected direction, but for some movements the association was weaker than anticipated (Table 3). As hypothesized, associations with SPADI generally seem stronger for measures of active ROM than for passive ROM, and stronger for the disability subscale than for the pain subscale.

**Table 3:** Correlation (*r*) of ROM and SPADI change after treatment (n = 75)

**Examined movement**	**Pain subscale**	**Disability subscale**	**SPADI total**
P. ABD	**-0.26**	**-0.28**	**-0.29**
P. FLE	-0.03	-0.09	-0.09
P. INT	-0.02	-0.15	**-**0.09
P. EXT	-0.15	**-0.33**	-**0.25**
C. PROM	-0.15	**-0.29**	**-0.23**
			
A. ABD	**-0.31**	**-0.32**	**-0.33**
A. FLE	**-0.30**	**-0.36**	**-0.35**
A. INT	-0.14	**-0.31**	-**0.23**
A. EXT	-0.22	**-0.33**	**-0.29**
C. AROM	**-0.32**	**-0.40**	**-0.38**

Results of the multiple linear regression analysis are given in Table 4. At first we also included variables for passive ROM (baseline and change for C.PROM), but these variables were omitted because the inclusion of these variables did not result in a significant improvement of the model. In the final model, 60% of the variance in SPADI improvement could be explained by variance in the independent variables, while only 40% could be explained if improvement in C.AROM was omitted from the analysis. Residuals were normally distributed and there was no evidence of heteroscedasticity [35].

**Table 4:** Results of linear regression analysis (n = 75)

	**B**	**SE of B**	**β**	**Sig.**
Constant	-40	11		0.001
Baseline SPADI	0.80	0.091	0.75	< 0.001
Baseline C.AROM	0.082	0.031	0.23	0.01
C. AROM improvement	0.13	0.022	0.46	< 0.001
				

SD = between-subject standard deviation, CI = confidence interval, SRM = standardized response mean, RCP = reliable change proportion.

## Discussion

The main finding in this study is that SPADI was more responsive than measurements of shoulder ROM. Comparing responsiveness of a self-evaluation questionnaire like SPADI and an impairment measure like shoulder ROM may seem odd to some readers. Few researchers have compared the responsiveness of shoulder scales with the responsiveness of traditional shoulder outcome measures. However, this type of comparison was a natural choice when investigating SPADI responsiveness in this population. Both SPADI and shoulder ROM have been used as outcome variables in several clinical trials involving patients with adhesive capsulitis. Furthermore, shoulder ROM was employed as a gold standard surrogate when testing criterion validity and responsiveness of SPADI in the original article by Roach et al. [1].

Reliability (reproducibility) is a necessary precondition for the appropriate application of change scores in general [42], but is especially important when interpreting change scores for individual patients [43]. Investigation of SPADI reproducibility with a test-retest design indicated that approximately 95% of the pairs of observations did not differ by more than 17 points. This is slightly better than what has previously been reported for other study populations where a similar design has been used [6,8,29].

Studying SPADI reproducibility, we observed a mean score difference between the two administrations of the questionnaire. The finding suggests some form of session bias. One could suspect that ROM measurements on the first test occasion caused temporarily increased pain in some patients. Another possible explanation is that patients were included early in the development of the condition, so that a stable pain situation had not yet been reached. Measurements in this study were analyzed according to a simple one-way ANOVA model, and thus we did not plan to control for a session effect. This means that our results might tend to over-estimate measurement errors. However, we analyzed data by a two-way ANOVA model post-hoc, and results for reproducibility were very similar to those reported in this study from the one-way model analyses.

Estimates for reproducibility were used in subsequent responsiveness analyses to produce a "reliable change proportion" (RCP) figure for SPADI which was compared with corresponding figures for shoulder ROM. A higher RCP was found for SPADI than for the individual movements of shoulder ROM. This could be interpreted either as smaller measurement errors or as a larger "change" for SPADI.

Group-level responsiveness was investigated by the "standardized response mean" (SRM) statistic. Again, the SPADI was more responsive than individual-movement ROM measures. The combined ROM measures were also generally more responsive than the individual-movement measures, the reason for this being the relatively smaller measurement errors for combined movements [32,44]. From purely statistical and practical points of view, the SPADI appears as a more attractive outcome measure than shoulder ROM in this study. The ratio of sample sizes required to detect a given clinical effect is equal to the square of the ratio of standardized response means [45]. Hence estimates for SRM indicate two- or three-fold increases in necessary sample size if a single ROM movement is chosen instead of SPADI as the primary outcome measure in a trial of this type. Likewise, if SPADI and a ROM measure are both used as outcome measures in a trial, SPADI may have a better chance than the ROM measure to detect a treatment effect. The possibility to detect a significant difference is one thing, however. When comparing responsiveness of ROM and SPADI in this way, we compare the ability to detect change, but we are not comparing the ability to detect the *same *change. We analyze the change in scales which do not measure the same construct. Clearly, the aim of each separate study must guide in the selection of outcome measures.

Compared to previous studies reporting SRM for SPADI, estimates in this population are high. Five previous studies report much lower SRMs [6,7,9,11,34], while in a recently published study [10], SRM for SPADI was almost as high as in this study. The researchers also reported responsiveness of the cASES "Motion active" (SRM: 1.54) and "Motion passive" (SRM: 1.47), which are constructs that (roughly) correspond to the constructs C.AROM (SRM: 1.28) and C.PROM (SRM: 1.52) in the present study. Responsiveness for these ROM measures is quite similar in the two studies, and in both studies lower than the SPADI total. However, results for responsiveness are in general specific to the study population, the intervention and the overall design of the study. Comparisons across studies are difficult.

In this study, confidence intervals and test statistics for the SRM and RCP are calculated as if the estimated variability in scores (SD of change and SDD, respectively) represents the underlying "true" variability. This is an approximation. A more exact method would probably result in wider confidence intervals and more conservative test results.

Investigation of "external" responsiveness is sometimes performed in order to demonstrate that a new measure may replace an old one. This was not really our aim: we simply wanted to investigate the relationship between changes in shoulder ROM and SPADI in these patients. Since there is no valid way to measure change directly [42], comparisons were based on the difference between respective measurements for the two different time points. Correlations between improvement in ROM and SPADI were below 0.50. In the original study by Roach et al. [1], associations between SPADI and active ROM change scores were stronger, coefficients ranging from -0.52 to -0.70. Limited information regarding the variability of change scores in that study restricts further comparisons, however.

The ability to discern associations among variables is impaired by a lack of reproducibility in direct proportion to the products of the reliabilities of the measurements involved [46]. Furthermore, one must expect the reliability of a difference between measurements to be lower than the reliability of the separate measurements [44]. Hence there is reason to believe that the association between "true" changes for ROM and SPADI is somewhat stronger than indicated by the correlation coefficients reported in this study.

Of the translation process, the Stage VI (submission of reports to the developers of the original questionnaire) was not performed, and this is a weakness of the study. We were not able to get in contact with the respective researchers.

## Conclusion

Results indicate that SPADI is more responsive than shoulder ROM measurements, which have been extensively employed as shoulder outcome variables in these patients. The relationship between changes in shoulder ROM and SPADI suggest that they measure overlapping underlying phenomena. The results in this study support incorporating the SPADI questionnaire in patient evaluation procedures when designing clinical trials where patients with adhesive capsulitis are investigated.

## Competing interests

The authors declare that they have no competing interests.

## Authors' contributions

All authors contributed to study design. EKT recruited the patients, performed the statistical analysis and drafted the manuscript. OME administered questionnaires and measured range-of-motion. OME, NGJ and EBH helped to draft the manuscript. All authors read and approved the final manuscript.

## Pre-publication history

The pre-publication history for this paper can be accessed here:



## Supplementary Material

Additional file 1 **Norwegian SPADI**. The Norwegian version of the SPADI questionnaire Click here for file
