# Supplementary material for: Responsiveness of the Shoulder Pain and Disability Index in patients with adhesive capsulitis
Source: BMC Musculoskelet Disord. 2008 Dec 3;9:161. doi: 10.1186/1471-2474-9-161 (PMC2633286; doi:10.1186/1471-2474-9-161)
Supplement: Additional file 1 — Norwegian SPADI. The Norwegian version of the SPADI questionnaire [file 1471-2474-9-161-S1.pdf]

SPADI

Shoulder Pain and Disability Index

Norsk versjon

Roach KE et al. **Development of a shoulder pain and disability index**. *Arthritis Care and Research* 1991, **4**(4):143-149.

Spørreskjema om vond skulder

Del I: Sett et merke på linjen for å vise hvor sterke SMERTER du har hatt **den siste uken** for hvert spørsmål

Eksempel:

Ingen smerter ——————X—————————— Verst tenkelige smerter

1. På det verste?

Ingen smerter ________________________________ Verst tenkelige smerter

2. Når du lå på den vonde siden?

Ingen smerter ________________________________ Verst tenkelige smerter

3. Når du strakte deg etter noe på en høy hylle?

Ingen smerter ________________________________ Verst tenkelige smerter

4. Når du tok på nakken?

Ingen smerter ________________________________ Verst tenkelige smerter

5. Når du skjøv med den vonde armen?

Ingen smerter ________________________________ Verst tenkelige smerter

Del II: Sett ett merke på linjen for å vise hvor store VANSKER du har hatt **den siste uken** når du utførte aktivitetene som er opplistet nedenfor

1. Når du vasket håret?

Ingen vansker ______________________________ Så vanskelig at jeg måtte ha hjelp

2. Når du vasket deg på ryggen?

Ingen vansker ______________________________ Så vanskelig at jeg måtte ha hjelp

3. Når du tok på deg undertrøye eller genser?

Ingen vansker ______________________________ Så vanskelig at jeg måtte ha hjelp

4. Når du tok på deg en skjorte med knepping foran?

Ingen vansker ______________________________ Så vanskelig at jeg måtte ha hjelp

5. Når du tok på deg buksene?

Ingen vansker ______________________________ Så vanskelig at jeg måtte ha hjelp

6. Når du plasserte en gjenstand på en høy hylle?

Ingen vansker ______________________________ Så vanskelig at jeg måtte ha hjelp

7. Når du bar en tung gjenstand på 5 kg eller mer?

Ingen vansker ______________________________ Så vanskelig at jeg måtte ha hjelp

8. Når du tok opp noe fra bak-lomma?

Ingen vansker ______________________________ Så vanskelig at jeg måtte ha hjelp
